# Comment on “Refining Auxiliary Orthotopic Liver Transplantation (AOLT) Improves Outcomes in Adult Patients With Acute Liver Failure”

**DOI:** 10.1097/AS9.0000000000000386

**Published:** 2024-02-19

**Authors:** Fabien Robin, Karim Boudjema

**Affiliations:** * From the Department of Hepatobiliary and Digestive Surgery, Pontchaillou University Hospital, University of Rennes 1, Rennes, France.

I was very pleased to read the paper on auxiliary partial orthotopic liver transplantation (APOLT) by the Beaujon group in Paris, recently published in *Annals of Surgery*.^1^ It reports on a large series of APOLs using a right graft and praises the advantages of this method over left APOLTs.

The paper, which is of high quality, suggests that the authors were the first to use a right hemigraft to compensate for the recipient’s severe liver failure. With this very respectful comment, I would like to set the record straight and remind that the first reported APOLT was by the team of Prof. Rudolph Pichelmayer.^2^ The postoperative period was so stormy that the method did not have the impact it deserved. The proof of concept of APOLT was then reported by the Strasbourg group. The effectiveness of left APOLT was demonstrated to such an extent that the observation was published in the *Lancet*.^3^

Also in Strasbourg, aware of the need to provide large quantities of liver to patients who were often on the verge of brain death, we were the first to perform right APOLTs. This pioneering experiment involved 3 cases.^4^ The graft retrohepatic vena cava was implanted end-to-side on the right side of the native inferior vena cava after it was exposed through a right hemihepatectomy, the graft portal vein was implanted on the enlarged ostium of the native portal trunk, and the graft artery was implanted on the native common hepatic artery or on the aorta. Eventually the graft common bile duct was diverted in a jejunal limb.

We regret that these pioneer papers have not been referenced so that readers of *Annals of Surgery* could be aware of it.
